# The impact of financial development on income inequality and poverty

**DOI:** 10.1371/journal.pone.0291651

**Published:** 2023-10-26

**Authors:** Ayşe Aylin Bayar

**Affiliations:** Department of Management Engineering, Economics Division, Faculty of Management, Istanbul Technical University, Istanbul, Türkiye; Usak University: Usak Universitesi, TURKEY

## Abstract

The Turkish economy has undergone a structural transformation with impressive economic performance during 2002–2018 and then a slowdown. The implementation of policies on the financial markets results in a significant capital inflow, which leads to an increase in the volume of domestic credit. Despite improvements in Türkiye, income inequality and poverty are still relatively high. While much of the literature shows that financial development accelerates growth, there is no consensus on its clear impact on poverty and inequality. While some studies stress that financial development improves inequality and combats poverty by increasing the ability of advantage of new investment opportunities, and by improving the allocation of capital, others point out that the beneficial impact of financial development depends on whether the overall population or the upper-income groups benefit or not. Therefore, this paper aims to empirically investigate the impact of financial development on inequalities and poverty during the 2002–2017 period when Türkiye relatively has been prosperous. According to simultaneous equation regression findings, the widening of the financial sector leads to more equal income distribution and poverty alleviation.

## Introduction

Several studies have investigated the causal relationship between financial development, (which is the extension of the banking and financial services sector), income inequality, and poverty for a long time and attempted to provide clear evidence on the effects of financial development on poverty and income inequality [1–10]. While there has been a great deal of literature on this issue, there is no consensus on the proposition that financial development is a remedy for damaged income distribution. It has been proven by some studies that a well-organized financial sector is the only way to make a positive contribution to macroeconomic indicators. Financial development can be used as a tool to reduce inequality and combat poverty in developing countries that are experiencing high inequality and poverty rates. That is why the impact of financial development is widely examined for developing countries in the literature.

There has been a stunning economic performance in the Turkish economy during 2002 and 2018 and there is a slowdown afterwards. Improvements have been observed in the main macroeconomic indicators of Türkiye. Despite witnessing these improvements, she is still experiencing some distributional problems. She has previously documented the worst income distribution and poverty among OECD countries [11–13], which makes the distributional problems especially critical. The main factors behind this poor distributional outcome are a persistently high inflation record, ongoing high-interest rate policies, and political and economic instability. After the 1990s, the Turkish economy has undergone structural and social transformation due to trade and financial liberalization. In that regard, macroeconomic policies have some distributional effects on income inequality and poverty. The complexity and persistence of dealing with inequality and poverty have led to ongoing debates among researchers about how to overcome these problems.

For Türkiye, unfortunately, there is a lack of data about inequality and poverty, and therefore, limited studies can examine this issue empirically before 2001. One of the well-known Gürsel et al. (2000) [12] studies argued that income inequality slightly increased from 1987 to 1994 in Türkiye. Some other studies focus on inequality and poverty in Türkiye before the year 2000 [14–16]. Although income distribution has improved after 2001, income inequality and poverty are still high. Therefore, researchers who focus on the period after 2001 analyze inequality and poverty by examining the distributional problems using different approaches. According to the researchers, financial development and the improvement of access to financial institutions play a crucial role in reducing inequality and fighting poverty. The objective of this paper is to examine the impact of financial development on inequality and poverty in the Turkish economy between 2002 and 2017. This linkage is extensively revealed through the use of a variety of econometric analyses.

The paper is organized as follows. Following section provides a summary of the studies on financial development, inequality, and poverty. The economic and financial indicators of the Turkish economy are given in Third Section. The data, empirical analysis, and findings are presented in Section 4, and Section 5. The conclusion is given in Section 6.

## Literature on financial development, inequality, and poverty

In particular, for developing countries, reducing poverty and improving income inequality have attracted a lot of attention from researchers. Macroeconomic policies to overcome such obstacles are one of the most debated questions in the literature. Thus, the impact of financial development is studied to tackle the problems of poverty and inequality.

The connection between financial development, inequality, and poverty holds special interest. The studies indicate that financial development, directly and indirectly, impacts income distribution and poverty [2, 7–10]. The findings of these studies demonstrate that financial development accelerates economic growth indirectly by mobilizing savings, diversifying risks, and improving entrepreneurs’ positions. It contributes to economic growth by fostering physical capital accumulation [1, 3–6, 9]. Furthermore, it is believed that economic growth, inequality, and poverty are strongly connected [17–20]. By increasing the average incomes of the poorest 20% of society proportionately, growth causes a change in poverty. It is revealed that policies that promote growth bring about benefits that are mostly in favor of the poor, which is known as pro-poor growth. If policies are pro-poor, their impact on reducing poverty will be much more significant. For instance, Appiah-Otoo and Song (2021) [21] question whether fintech and its sub-measures reduce poverty in China or not and their findings show that fintech complements economic growth and financial development in the country and therefore, helps to reduce poverty.

One of the most recent papers by de Hann et al. (2022) [22], studies the both indirect and direct link between financial development and poverty by utilizing a large panel of 84 countries over the period 1975 to 2014. The findings suggest that financial development does not directly reduce the poverty gap (or headcount poverty), but there are indirect effects, in which lower income inequality reduces poverty, but there is no effect on economic growth and financial instability. According to the results, financial development indirectly impacts poverty adversely as it leads to an increase in income inequality. These findings exhibit that the overall effect of financial development on poverty results positively or negatively, depending on which indirect effect, i.e. that of income inequality or growth, is stronger. Moreover, Demir et al. (2020) [23] develop a model for the interrelationship between Fintech, financial inclusion, and income inequality for a panel of 140 countries and suggest Fintech has an impact on inequality directly and indirectly through financial inclusion and the effects of financial inclusion on inequality are primarily associated with higher-income countries.

Additionally, Chisadza and Biyase (2023) [24] demonstrate the impact of financial development on income inequality for a global sample of countries (advanced, emerging, and developing) between 1980 and 2019. The findings reveal a positive impact of financial development on inequality for emerging and least-developed countries, but they can not find a significant conclusion for advanced countries. They further disaggregate the financial development into financial institutions and financial markets and they demonstrate that while the development of the banking sector leads to an improvement in income inequality in emerging and least developed countries, the development of the stock market adversely affects inequality in the least developed countries.

Other studies question whether financial development is beneficial to everyone in society or not. Some have concluded that developing countries’ financial development improves inequality and reduces poverty [7, 25–27]. This is due to the development of financial markets that have a more positive impact on developing countries than on developed countries, as freer markets strengthen lending capacity. Therefore, it facilitates the poor in investing in both physical capital and human capital. By widening financial opportunities, the poor can invest and equalize income distribution [28, 29]. A similar conclusion is obtained for Australia in the work of Shi et al. (2020) [30] where the impact of financial deepening on income inequality is revealed. According to the obtained findings, financial development indicators have a significant positive impact on income inequality.

Despite studies emphasizing a positive connection between financial development, inequality, and poverty, several other studies point out the opposite. These studies indicate that the poor lack sufficient access to credit during the development of the financial sector due to their position. According to empirical studies from developing countries and cross-country cases, those who are better off in society are more likely to benefit from new financial opportunities in proportion. This widens the gap in the income distribution of society and worsens the poverty rate [31, 32]. For instance, a recent paper, that examines emerging countries, suggests that even though financial development improves economic growth, this improvement does not necessarily benefit the ones on low-income groups, and also financial development has no significant role in poverty alleviation in these countries [33]. Besides, Sethi et al. (2021) [34], assert financial development and globalization deteriorates income inequality in India. Moreover, some studies also indicate that high-income groups and people with political connections primarily benefit from financial development which can lead to a volatile macroeconomic environment. As a result, it worsens inequality and raises the poverty rate [35–37].

A recent study proves that as a developing country, China is not passing the turning point of the inverted U-shaped curve yet, and therefore, the empirical findings show that financial deepening worsens inequality [38]. In addition, a similar approach is applied in some studies which predict an inverted U-shaped relationship between financial development and inequality [39, 40]. The idea of such a relationship is based on Kuznet’s (1955) [41] work on economic development and inequality. According to him, at the early stage of development, while the importance of the agricultural sector in the economy declines, income inequality worsens and later slows down with the development of the industry and improves with the increased percentage of the service sector. Therefore, the linkage between development and inequality is inverted U-shaped. As a result, studies that hypothesize a reverse U-shaped association between financial development and inequality are an extension of Kuznet’s idea. It is mentioned that financial development results in economic development and this has a significant impact on the distribution of income.

Extensive research suggests different empirical findings for countries. Cross-country cases, panel data, and country-specific studies have been employed to observe the linkage between financial development, inequality, and poverty [7, 25, 33–46]. For the Turkish economy, there exist limited empirical studies, which mainly suggest a positive effect of financial development on inequality and poverty [47–55]. For instance, while Kar et al. (2011) [50] empirically examine the relationship between financial development and poverty alleviation in Türkiye and conclude that financial development has a limited effect on poverty reduction through economic growth, Koçak and Uzay (2019) [51] emphasize the impact of financial development on inequality and suggest that it has a significant impact in the long term. Furthermore, Destek et al. (2020) [52] discuss the impact of financial development on inequality, whether it has a U-shaped shape or not. Their findings suggest that in the early stages of economic development, inequality is negatively impacted by financial development. The availability of credit for lower-income groups later on improves income inequality. Another recent study examines the impact of financial inclusion on poverty in Türkiye and points out that an increase in financial inclusion leads to a decrease in poverty [53].

Besides, similar to the other studies, Cetin et al. (2021) [54] conclude that financial development has a positive impact on income inequality for Türkiye, as well. A close idea and methodology with this paper is employed by Calis and Gökçeli (2022) [55], and they develop a VAR and Granger causality model to analyze the impact of financial inclusion on income inequality in Türkiye and like the other studies in the literature, they find that financial inclusion cause an improvement in the inequality and there is a unidirectional causality is found from financial inclusion to income inequality.

## Economic and financial indicators for Türkiye

During the 1990s, the Turkish economy experienced underwhelming macroeconomic and financial indicators. During that time, she faced unexpected fluctuations, political instability, a large budget burden, a trade deficit, high interest rates, inflation, and unemployment rates. At the end of this period, she was hit by a severe economic crisis in 2001. With the agreement of the International Monetary Fund, she began to implement solid macroeconomic policies, which included fiscal and monetary policies. Her economic performance after 2001 was spectacular, and the reform package had a positive impact on the overall economy. Most macroeconomic indicators improved and rates declined. After 2001, there was a period of high economic growth until the global economic crisis that resulted from the American subprime mortgage crisis in 2008. Even though there has been improvement in macroeconomic indicators, as a small economy, she was still vulnerable to international fluctuations, which is why the global crisis has hit negatively. To examine more closely, a summary of Turkish macroeconomic and financial conditions is discussed in this section.

Table 1 includes the data that is derived from the Ministry of Development, Economic and Social Indicators, The Central Bank of the Turkish Republic, and the Turkish Statistical Institute. The table indicates that there has been economic growth (7 percent) in the Turkish economy between 2001 and 2008 which was a steady recovery from negative GDP growth in 2001. This growth was followed by a contraction in 2008 when a global financial crisis negatively affected the economy. Following this period, there has been another improvement, and the growth rate has been around 6% and 4% from 2010 to 2013, and from 2013 to 2017, respectively. Indeed, these figures reveal that, as a small country, the Turkish economy is affected by the global era and fluctuations. Until 2000, inflation and interest rates were high, and they reached their peak in 2002. Until the period of 2013–2017, these rates declined to 7.6% and 11.1%, respectively, but then went up to 8.8% and 10.2% in the 2013–2017 period. Easy access to international financing and easy utilization of financial resources have resulted in relatively low rates during this period.

**Table 1:** Main macroeconomic and financial indicators.

	1995–2000	2001	2002–2007	2008–2009	2010–2013	2013–2017
	%					
**GDP Growth**	4.7	-5.7	6.8	-2.1	6.0	4.4
**Inflation Rate**	69.5	68.5	12.8	8.1	7.6	8.8
**Interest Rate**	74.4	62.5	26.0	14.3	15.1	10.2
	% *of GDP*					
**PSBR**	7.3	12.1	3.2	3.4	1.0	0.5
**Current Account Balance**	-1.1	1.9	-3.9	-4.0	-7.5	-2.7
**Money Supply (M2)**	11.7	16.4	24.6	49.0	53.6	54.7
**Private Banks Credits**	13.1	0.8	15.7	29.9	47.3	57.1
**Total TL Deposits**	15.2	18.2	20.5	31.5	35.4	32.8
	% *Change*					
Real Effective Rate*	7.6	-21.2	9.0	-5.2	0.4	-6.9
**Nominal Exchange Rate**	66.6	96.5	1.5	9.5	5.4	15.5
**Nominal Wage Index**	77.3	31.9	17.8	8.3*	7.9^*^	10.8*

* Until 2008, the Nominal wage index was taken from Economic and Social Indicators, and then in recent years, the nominal wage index for the manufacturing sector became available from TurkStat.

The implementation of a sound fiscal policy by policymakers resulted in a decline in the public sector borrowing requirement (PSBR) as a percentage of total income until 2008. Similarly, to other macroeconomic indicators, it increased after the global financial crisis in 2008 reached 4%, and decreased to an average of 0.5% between 2013 and 2017. Structural reforms and privatizations have caused a decline in the PSBR. As a result, the public’s role in the economy changed while the private sector began to gain importance. Economic growth was sustained with the increase in productivity in the private sector.

The nominal exchange rate and real effective rate have the same trends: there was a decline during the 2001–2008 period, but a rise in the year 2008. In 2008, the nominal exchange rate reached its lowest level of 1.299 TL, and then it attempted to increase to control the current account deficit. Table 1 shows that the nominal exchange rate and the real effective rate increased between 2013 and 2017. There is no doubt that all macroeconomic indicators have improved after 2001, except for 2008, until 2013. However, the positive atmosphere seems to have deteriorated from 2013 to 2017.

Private bank credits, total deposits, and money supply as a share of total income can be used to measure financial development or depth. These figures take into account the growth of the financial services industry. The financial sector’s development allows savings to be channeled into efficient investment opportunities, resulting in increased capital accumulation and economic growth. Table 1 data shows that all financial indicators are experiencing a parallel trend of increasing over the entire period. To create strong macroeconomic indicators in the economy, money supply, bank deposits, and domestic credits are employed to mitigate the deterioration effects of the crisis in 2001. Between 2002 and 2017, financial depth was achieved by increasing the average share of money supply, domestic credit, and bank deposits.

Table 1 summarizes that the Turkish economy experienced distinct conditions in several sub-periods after 2001. During the first period, from 2002 to 2007, when the reforms were implemented, overall macroeconomic and financial indicators improved. With the boom of the international era, the inflow of foreign capital to Türkiye has accelerated, and sound macroeconomic policies have created opportunities for foreigners to invest. Depending on the positive developments in the international financial markets, foreign funds were used to finance economic growth in that sense. This situation has resulted in a growth process that is driven by domestic demand, with the domestic private sector obtaining long-term funds from abroad at a low cost. With the restructuring of the banking system and the independence of the Central Bank, the burden and constraints on foreign investment have been reduced and foreign capital investment was boosted by new legal regulations.

However, these optimistic conditions were reversed by the subprime mortgage crisis of 2008. Therefore, the period of 2008–2009 is given separately in the table. Even though this crisis appears to be a financial one, at first, the financial institution issues deteriorated economic activities and investments, leading to a global recession. The impact of this financial crisis on the economic indicators could be captured by the period between 2010 and 2013. As a result of financial crisis there occurred a decline in capital inflows from foreign investors, and the Turkish government has tried to sustain economic growth with a domestic credit boom and an increase in public expenditure during this period. At the same time, the shares of financial indicators in GDP reveal that financial development and financial depth can be achieved over the same period. And then after 2013, Türkiye implemented several recovery policies to overcome the problems in the economy and therefore, the period of 2010 and 2013 is given separately in Table 1. As observed from the table, even though there is a slowdown in economic growth, financial indicators such as private bank credits and total TL deposits are improved.

**Fig 1:**
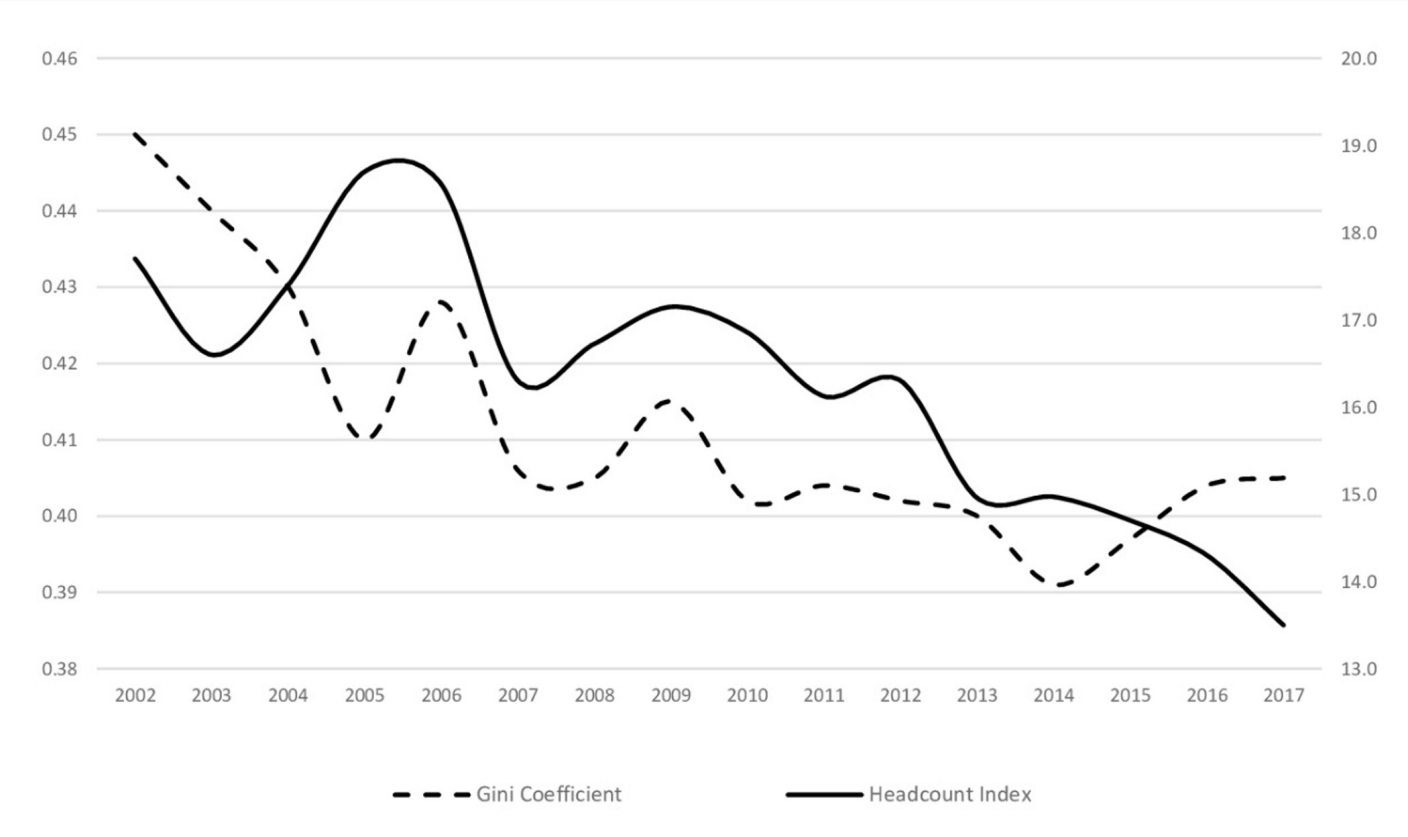
Income distribution and poverty in Turkey is about here.

Although macroeconomic and financial indicators indicate these improvements, the Turkish economy exhibits interesting distributional results that are represented by the Gini coefficient, a measure of inequality; and the headcount ratio, a measure of poverty in Fig 1. In this figure is for 2002–2006 the data is derived from Household Budget Surveys, and for 2006–2017, it is derived from the Survey of Income and Living Conditions. It should be noted that section 4 provides detailed information about the Gini coefficient and Headcount index, which are the most well-known measures for income inequality and poverty.

It should be noted that section 4 provides detailed information about the Gini coefficient and Headcount index, which are the most well-known measures for income inequality and poverty.

Fig 1 indicates that both measures followed two different trends after 2002. First, the rapid decline between 2002 and 2007 indicates an improvement in income inequality. In 2002, it was 0.45 and then decreased to 0.41 in 2007. After 2007, it fluctuated and eventually formed a smooth line between 2009 and 2013. However, when analyzed more closely, despite the improvement in income distribution, there is not a significant difference between statistics. This indicates that the distributional policies were not enough to overcome unequal distribution. When poverty rates are examined, it seems that the Turkish economy is slightly better in terms of poverty rates than income inequality. The number of people who remain below the predetermined poverty line has a declining trend and continues to fall below 13%. In 2002, it was 17.7%, but it dropped almost 5 points throughout the investigated years.

**Fig 2:**
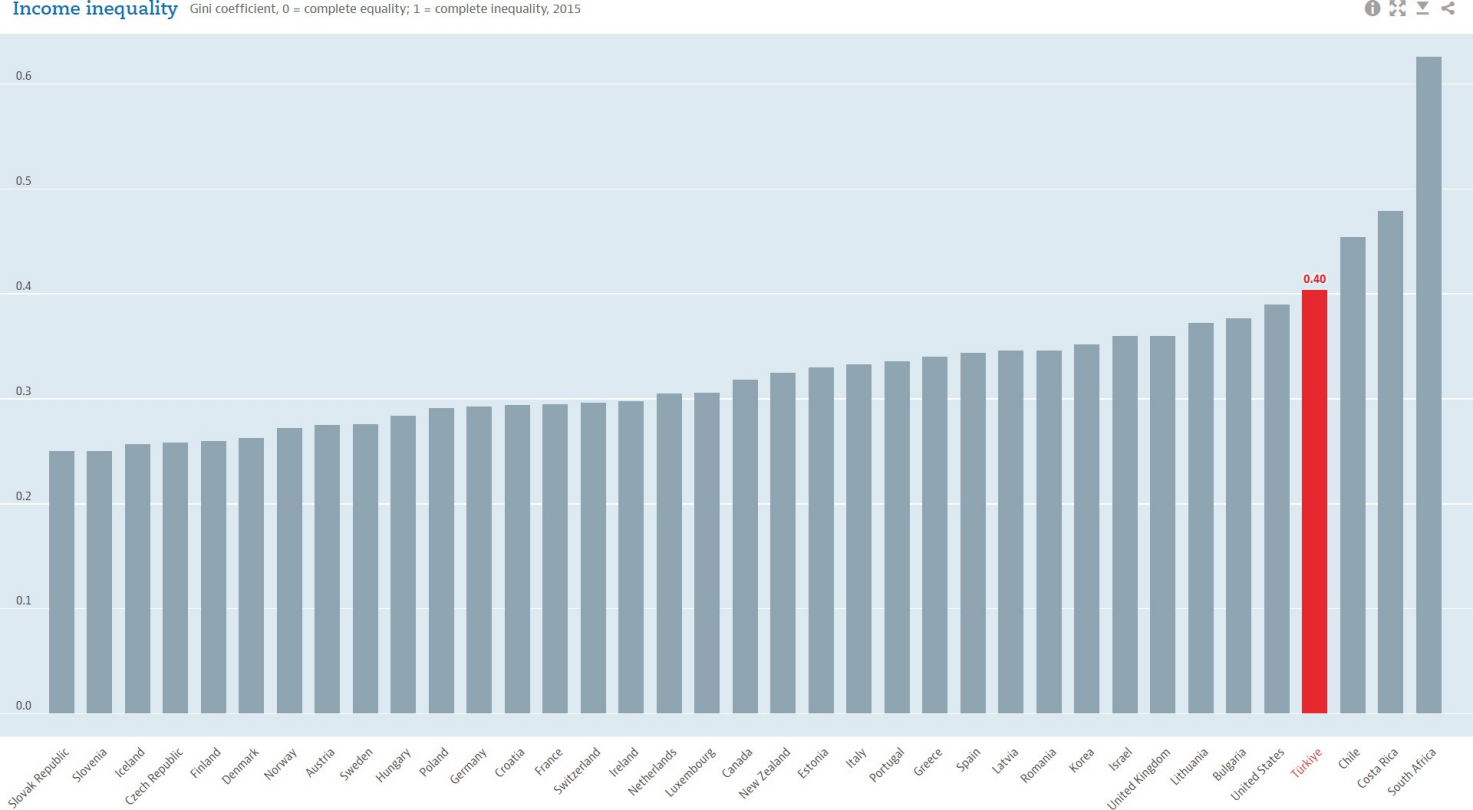
Gini coefficients for OECD countries is about here.

**Fig 3:**
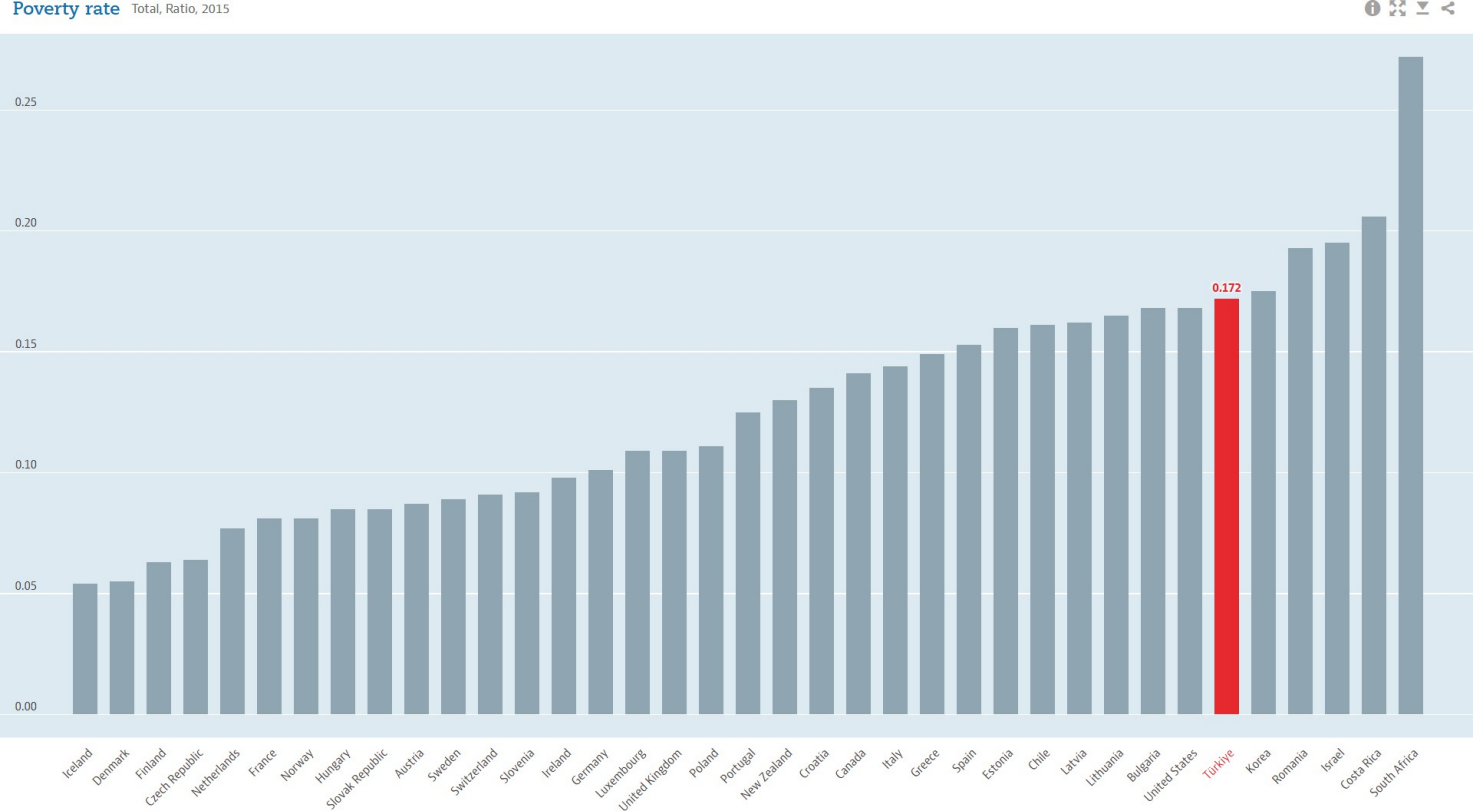
Poverty-rate-for-OECD-countriesis about here.

The Gini coefficients and poverty rates in OECD countries are depicted in Figs 2 and 3 [56]. As seen in Fig 2, Türkiye, which is highlighted in the red column, remains one of the worst countries for inequality and poverty in OECD countries over this period. Chile, Costa Rica, and South Africa were the only countries where her income distribution was better than in 2015.

The poverty rates exhibit the same pattern. The headcount index represented in the Fig 3, is the ratio of the number of individuals whose income falls below the poverty line and is taken as half the median household income of the total population. The Turkish economy has a headcount index that is higher than most of the OECD countries. Poverty in Türkiye was around 17% in 2015. The vulnerability of the economy is represented by the poor who live under a pre-determined poverty line. Therefore, the policy’s primary objective is to alleviate poverty, or at least reduce it. As seen from the Fig 3, although the Turkish poverty rate is not the highest in OECD countries, it is still prominent as it doesn’t decline as desired.

## Data and methodology

The main purpose of this paper is to explore the effects of financial development on inequality and poverty. To achieve this aim, the data taken from the World Development Indicators and Global Financial Development databases of the year 2019 are analyzed. A simultaneous equations model is utilized to reveal the connection between financial development, poverty, and inequality. Since there is correlation and causality among these variables, ordinary least squares estimates are not employed for satisfying consistent and non-biased estimates of model parameters as in the simultaneous equations, a dependent variable in one equation is an explanatory variable in another. This model can handle a potential endogeneity problem. Below is a representation of the poverty equation, growth equation, and inequality equation. In these models, control variables are added to variables such as trade openness, government spending, inflation, and population growth.

$$P_{t}=\;\beta_{0}\;+\;\beta_{1}GDP_{t}\;+\;\beta_{2}\;Gini_{t}\;+\;\beta_{3}FD_{t}\;+\;+\;\beta_{5}\;Pop_{t}\;+\;\varepsilon_{t}$$ (1)

$$GDP_{t}\;=\;\alpha_{0}\;+\;\alpha_{1}\;Gini_{t}\;+\;\alpha_{2}FD_{t}\;+\;\alpha_{3}T_{t}\;+\;\alpha_{4}G_{t}\;+\;\alpha_{5}l_{t}\;+\;\varphi_{t}$$ (2)

$$Gini_{t}\;=\;\gamma_{0}\;+\;\gamma_{1}GDP_{t}\;+\;\gamma_{2}GDP_{t}^{2}\;+\;\gamma_{3}FD_{t}+\varepsilon_{t}$$ (3)

where P_t_ represents the headcount index, GDP_t_ is the growth rate of total income, FD_t_ is the proxy variable for financial development, T_t_ is trade openness, I_t_ is the inflation rate, G_t_ is government spending, and GINI_t_ is income inequality.

According to the studies, the ratio of private credit to total income (GDP), the ratio of bank liquid reserves to bank assets, and the ratio of domestic credit of the banking sector to total income are generally used as proxies for financial development. In the literature, for developing countries, the ratio of private credit to total income is commonly chosen. That is why in this paper, this variable is employed, as well. And, to find out accurate results, it is assumed that financial development is the only common explanatory variable in all equations. The literature indicates that financial development is likely to have n simultaneous effects on the three endogenous variables [42].

As previously mentioned, there are a number of common measures for poverty and inequality in the literature. The most well-known ones are utilized for empirical analysis as indicators of poverty and inequality. The common poverty measurements are the measures of the Foster-Greer-Thorbecke (FGT) class of poverty, namely the headcount ratio (P0), the poverty gap (P1), and the squared poverty gap (P2) [58, 59]. The general formula for the FGT class of poverty measures takes different names depending on a parameter α which is zero for the headcount index, one for the poverty gap, and two for the squared poverty gap. The formula can be expressed as follows:

$$P_{\alpha }={\frac{\sum_{i=1}^{q}\left(1-\frac{y_{i}}{z}\right)}{N}}^{\alpha }$$ (4)

where z is the poverty line, (1-_Yi_) is the poverty gap, N is the size of the sample, and α is a parameter. For the empirical analysis, the head-count index (P_0_) which is the ratio of the population whose income level is lower than the pre-determined poverty line, is chosen as a proxy of poverty.

Besides, among some common different measures, which are employed to express the income distribution the most well-known measure, the Gini coefficient is preferred in this paper distribution [57–59], The Gini coefficient can be written as follows:

$$\text{Gini}\;=\frac{1}{2n^{2}\bar{y}}\left[\sum_{i=1}^{n}\sum_{j=1}^{n}|y_{i}-y_{j}|\right]$$ (5)

where *n* is the number of individuals, y_i_ and y_j_ are individuals’ income level, *i* ∈ (1, 2, 3, … ,*n*), and $\bar{y}$ is the arithmetic mean income. The Gini coefficient takes value from “0” to “1”. If the distribution of incomes is completely equal (unequal), the Gini index is equal to zero (one).

In addition, control variables are included in the equations. First, the growth rate of GDP per capita is used as a proxy for growth. Secondly, the degree of openness is revealed by adding the sum of exports and imports as a share of GDP. Third, the government’s role in economic activities is captured through the inclusion of government spending. It is believed that the government’s role decreases with increased economic growth and reduced inequality.

Aside from simultaneous regression models, causality tests and the correlation between variables are utilized as well for the empirical analysis. For causality control, the Granger Causality test is applied. The main purpose of this test is to check the direction of the relation between the variables. Granger’s causality is a statistical test to determine if a time series is useful for forecasting another time series. Granger defined causality as “If the prediction of Y is more successful when the past values of X are used, then X is the Granger cause of Y”, after testing the accuracy of this statement the relationship is shown as *X* → *Y* [60].

## Results

Before utilizing the simultaneous equation regression model to examine the impact of financial development on inequality and poverty, first, the correlation matrix for the variables in the regression model is utilized. The results are shown in Table 2. As observed from the table, there is a negative correlation between the private credits of banks as a share of GDP and the Gini coefficient. The degree of correlation between them is high, close to 1. This is also true for the headcount index. The correlation pattern of their correlation is very similar to that of the Gini coefficient. The correlation results exhibit the connection between financial development, inequality, and poverty. The correlation between financial development and poverty and inequality is negative. These results indicate that the increase in financial services development indicates a decline in inequality and poverty. These findings are compatible with the expectations. In addition, there is a positive correlation between financial development and growth. The more developed the financial services industry, the greater the growth of the economy.

**Table 2:** The correlation matrix.

	Private Cr. of Banks (%GDP)	Gini Coefficient	Headcount Ratio	Trade Openness (% GDP)	Inflation Rate (%)	Population Growth	GovernSpending (%GDP)	GDP growth
**Private Cr. of Banks (%GDP)**	1.000							
**Gini Coefficient**	-0.827	1.000						
**Head Count Ratio**	-0.874	0.656	1.000					
**Trade Openness (% GDP)**	0.581	-0.542	-0.536	1.000				
**Inflation Rate (%)**	-0.454	0.719	0.179	-0.189	1.000			
**Population Growth**	0.460	-0.524	-0.325	0.128	-0.253	1.000		
**Govern. Spending (% GDP)**	-0.356	0.559	0.131	-0.389	0.753	0.053	1.000	
**GDP per capita growth**	0.109	-0.215	0.023	0.296	-0.545	-0.384	-0.666	1.000

It is also observed that government spending as a share of GDP and Private Credit of Banks as a share of GDP correlate negatively. Another significant finding in correlations between variables is between inequality and growth. Growth can lead to improvements in inequality, as evidenced by a negative correlation. The correlation between growth and poverty reduction is weak, in contrast. And as expected, there is a negative correlation between the inflation rate and private bank credits as a share of GDP. The inflation rate will increase in the short run as financial services with lower credit rates and easy accessibility are expanded.

The Granger Causality test results are represented in Table 3. The results indicate that the lag level is two (2). A standard Chi-squared test for Granger causality was performed on all of the lag-length specifications. Then, Granger’s two-way causality is performed to check whether there is a causal direction from one to another. As observed from the table, the p-value (0.011) falls below the statistically significant threshold of 0.05 hence, the null hypothesis that lags of financial development variable does not impact the inequality is rejected. This indicates financial development is a Granger cause of inequality. The same holds for inequality and financial development. The null hypothesis is rejected because the p-value (0.058) does not meet the statistically significant threshold of 0.10. Therefore, the result shows that there is a two-way direction causality between financial development and inequality. In addition, there is also a two-way direction between poverty and inequality. The null hypotheses are not rejected as well. There is a one-way direction causality between the headcount index and financial development.

**Table 3:** Granger causality results.

Causality	Lag Values	p-value
Private Credit of Banks (%GDP) → Gini Coefficient	2	0.011*
Private Credit of Banks (%GDP) → Headcount Index	2	0.000*
Gini Coefficient → Private Credit of Banks (%GDP)	2	0.058**
Gini Coefficient → Headcount Index	2	0.100**
Headcount Index → Gini Coefficient	2	0.000*
Headcount Index → Private Credit of Banks (%GDP)	2	0.572

(*: statistically significant at %5, ** statistically significant at %10)

The obtained findings reveal that financial development is the Granger cause of the headcount index, whereas the headcount index is not the Granger cause of financial development. These empirical results are consistent with the findings of other studies in the literature. The findings validate theoretical expectations about the impact of financial development on income inequality and poverty in the Turkish case.

Table 4 presents the empirical findings of the simultaneous equations regression model. According to the results, GDP per capita growth has a positive impact on poverty. The arguments of Dollar and Kraay (2001) [17], which suggest an increased growth rate induces a lower poverty rate, cannot be met. Therefore, the poverty rate in Türkiye has worsened due to economic growth. This could be a result of the short time of the examination, and besides, during this period, the Turkish economy is undergoing a structural transformation and shifting economic priorities. The quality of growth is a crucial factor in poverty reduction, which results in pro-poor growth. According to this result, Türkiye’s growth is not sufficient to alleviate poverty. In the empirical model, inequality has no significant impact on poverty.

**Table 4:** Simultaneous equations regression findings.

	Poverty	Growth	Inequality
**Private Credit of Banks (%GDP)**	-0.080* (0.011)	0.617* (0.002)	-0.085* (0.000)
**Gini Coefficient**	.0.033 (0.411)	10.682* (0.001)	—
**GDP per capita growth**	0.020** (0.031)	—	-0.114 (0.126)
**GDP per capita growth (square)**	—	—	0.014 (0.191)
**Population Growth**	8.050** (0.087)	—	—
**Trade Openness**	—	1.389* (0.027)	—
**Inflation Rate**	—	-1.105* (0.004)	—
**Government Spending (%GDP)**	—	-0.571 (0.414)	—
**Constant**	5.744 (0.803)	-0.496* (0.002)	44.506* (0.000)

(*: statistically significant at %5, ** statistically significant at %10)

The table shows that financial development has a significant negative impact on poverty reduction in the Turkish case. The poverty rate is reduced by the development of financial services. This result is consistent with the expectations. Sustaining easy accessibility to financial services leads to a reduction in poverty. Besides, in some countries such as Türkiye, the poor have limited ability to access financial services, and when it improves, these groups will benefit more than other groups with financial development. So that leads to a lower poverty rate.

The third column of the table includes information on the impact of financial development on inequality. This finding is not once again surprising as it meets theoretical expectations. The inequality equation gives a negative statistically significant effect of financial development on inequality. This provides a fact that as financial services develop, the income distribution improves and the gap between the highest income group and the lowest income group narrows. However, the findings of the inequality equation do not support the Kuznets hypothesis, as the results of GDP per capita growth and its square are insignificant.

Furthermore, the growth model from the empirical analysis shows that financial development has a significant positive effect on growth. The other explanatory variables such as inflation rate, trade openness, and Gini coefficient are statistically significant. With greater trade openness, economic growth is accelerating, while rising inflation has resulted in lower growth. The theoretical assumptions are all consistent with these. According to the theoretical studies in the literature, financial development leads to a rise in growth by encouraging savings and diversifying risks.

## Conclusion

The primary objective of this paper is to examine the impact of financial development on inequality and poverty. Even though this question is commonly discussed in the literature, no consensus exists about the impact of financial development on inequality and poverty. While some studies claim that financial development leads to a reduction in poverty and improves income inequality, others argue the opposite. According to studies that provide the positive impact of financial development, widening financial services in the economy leads to the poor accessing more opportunities for finance. And it results in a more equitable distribution of income and a decrease in poverty rates. And, the development of financial services has an impact on poverty and inequality in two ways: directly and indirectly. Economic growth is the indirect consequence of financial development. It is stressed that financial development leads to economic growth and higher economic growth which in turn alleviates poverty and improves inequality.

As a developing country, Türkiye has experienced different economic conditions after 2002, and financial and economic stability are prominent key factors for sustainable growth, improvement in income inequality, and poverty alleviation. In that sense, exhibiting the role of financial development on these issues provides necessary information for policymakers. Even though the impact of financial development on inequality and poverty attracts attention a lot in the literature, there are limited numbers of studies on the Turkish case. Therefore, the findings of this paper not fulfil this gap in the literature, but also provide an opportunity to compare the findings with the other studies that focus on the Turkish economy.

With this respect, several empirical analyses are employed to demonstrate the relationship between financial development, poverty, and inequality in this paper. At first, correlation matrices and the Granger Causality test are utilized. According to the correlation matrices, financial development, poverty, and inequality are negatively correlated. Moreover, Granger Causality findings also support theoretical expectations and it is found that financial development is Granger cause of poverty and inequality.

Simultaneous equation regression models are employed to provide explicit results. As the endogeneity problem is overcame with these equations, consistent and non-biased results are obtained. The empirical findings are consistent with the theoretical assumptions. Empirical evidence suggests that financial development has a positive impact on poverty and inequality and therefore, for Türkiye, financial development helps to maintain more equal distribution and lower poverty rates. It is concluded that the development of financial services leads to poverty alleviation and improvement in the income inequality as it allows an enhancement for the limited access of the poor to financial services.

When the findings of this paper are compared with the previous literature about Türkiye, it is clear that the obtained results are consistent with the previous researches. Besides, this paper is not only consistent with the theoretical expectations and previous empirical studies about Türkiye, but also as it focuses on a long period, it gives an opportunity to examine the relationship between financial development, inequality, and poverty for the last two decades. In addition, this paper contributes to the literature by employing a methodology which is not utilized for the Turkish economy before. In that sense, compared to the other studies, this paper is the first one that used simultaneous equations for the Turkish case. For further research, to make a comparison with the empirical findings, in the model, different inequality and poverty measures could be utilized, and also to reveal the impact of financial development on inequality and poverty in more detail, regional disparity could be considered.
